# MCM6 indicates adverse tumor features and poor outcomes and promotes G1/S cell cycle progression in neuroblastoma

**DOI:** 10.1186/s12885-021-08344-z

**Published:** 2021-07-07

**Authors:** Yaoyao Gu, Xiaoxiao Hu, Xiaowei Liu, Cheng Cheng, Kai Chen, Yeming Wu, Zhixiang Wu

**Affiliations:** 1 Department of Pediatric Surgery, Xinhua Hospital, School of Medicine, Shanghai Jiaotong University, Shanghai, 200092 China; 2 Division of Pediatric Oncology, Shanghai Institute of Pediatric Research, Shanghai, 200092 China; 3 Department of Pediatric Surgery, The Second Affiliated Hospital and Yuying Children’s Hospital of Wenzhou Medical University, Wenzhou, China; 4 Department of Pediatric Surgery, Children’s Hospital of Soochow University, Suzhou, 215003 China

**Keywords:** MCM6, Neuroblastoma, Cell cycle

## Abstract

**Background:**

Minichromosome maintenance complex component 6 (MCM6), as an important replication permission factor, is involved in the pathogenesis of various tumors. Here we studied the expression of MCM6 in neuroblastoma and its influence on tumor characteristics and prognosis.

**Methods:**

Publicly available datasets were used to explore the influence of the differential expression of MCM6 on neuroblastoma tumor stage, risk and prognosis. In cell experiments, human neuroblastoma cell lines SK-N-SH and SK-N-BE [ (2)] were utilized to verify the ability of MCM6 to promote cell proliferation, migration and invasion. We further explored the possible molecular mechanism of MCM6 affecting the phenotype of neuroblastoma cells by mutual verification of RNA-seq and western blotting, and flow cytometry to inquire about its potential specific roles in the cell cycle.

**Results:**

Through multiple datasets mining, we found that high expression of MCM6 was positively correlated with elevated tumor stage, high risk and poor prognosis in neuroblastoma. At the cellular level, neuroblastoma cell proliferation, migration and invasion were significantly inhibited after MCM6 was interfered by siRNA. Mutual verification of RNA-seq and western blotting suggested that the downstream cell cycle-related genes were differentially expressed after MCM6 interference. Flow cytometric analysis revealed that neuroblastoma cells were blocked in G1/S phase after MCM6 interference.

**Conclusion:**

MCM6 is considered to be the driving force of G1/S cell cycle progression, and it is also a prognostic marker and a potential novel therapeutic target in neuroblastoma.

**Supplementary Information:**

The online version contains supplementary material available at 10.1186/s12885-021-08344-z.

## Background

Neuroblastoma is the most common extracranial solid tumor in infants, with 25–50 cases per million individuals [1]. It originates from the sympathetic nervous system. The incidence of neuroblastoma accounts for 8–10% of childhood tumors, and its fatality rate accounts for 15% of childhood tumor-related fatalities [2]. Neuroblastoma is highly heterogeneous both in phenotype and clinically, and its results range from maturity or spontaneous regression to aggressive progression [3]. The treatment strategy in neuroblastoma is determined by risk classification. Low-risk diseases usually resolve spontaneously, and satisfactory results can usually be obtained only through clinical observation or surgical resection. For moderate risk, the treatment plan is dependent on the response. The course of conventional chemotherapy is 4 to 8 cycles, usually at a lower dose than the high-risk regimen, and the primary tumor is surgically removed when possible. Owing to intensive treatment of high-risk tumors, surviving patients often suffer from multiple sequelae [4], and the recurrence rate of high-risk cases is as high as 50%, and once it recurs, it will be difficult to cure [5]. Selective inhibitors of cancer-specific abnormal pathways offer the opportunity to replace these conventional chemotherapy or reduce the dose required for therapeutic effects, thereby reducing the toxic side effects of high-risk neuroblastoma treatment.

DNA replication is a prerequisite for normal cell division. Therefore, abnormal DNA replication will drive the abnormal gene phenotype of the cell and further lead to malignant transformation [6]. Evidence has shown that MCM family proteins act as helicases in the initial stage of DNA replication and are key regulators of cell cycle checkpoints [7]. The MCM2–7 complex is initially loaded on the chromatin to initiate DNA unspool [8]. The binding of MCM6 to Cdt1, another component of the pre-replication complex, is the key to promoting the loading of the MCM complex on the chromatin for replication permission [9], so it is a potential marker for tumor diagnosis and treatment.

MCM6 is a member of the mini-chromosome maintenance family (MCM), which plays an important role in limiting the replication of each cell cycle [10]. MCM has identified at least 10 homologues in humans. Among them, the MCM2–7 complex participates in the formation of the pre-replication complex, and has helicase activity to unwind DNA, which leads to the recruitment of DNA polymerase and the initiation of DNA replication and extension [11]. MCM6 is a candidate marker for cell proliferation, and an increase in MCM6 levels indicates the proliferation of malignant cells. More and more evidence shows that MCM6 can predict tumor progression and prognosis. It is pointed out that MCM6 is abnormally expressed in a variety of malignant tumors, including liver cancer [12], non-small cell lung cancer [13], breast cancer [14] and cervical cancer [15]. In neuroblastoma, studies have reported that the MCM complex is directly regulated by the transcription factor MYCN [16], but there are very few studies on the tumor characteristics and prognosis of MCM6 and neuroblastoma. We boldly hypothesize that in neuroblastoma, MCM6 may promote the proliferation and poor prognosis of neuroblastoma by regulating the cell cycle process of neuroblastoma. Effective intervention of MCM6 is likely to become an effective new treatment for neuroblastoma.

## Methods

### Patients and tissue samples

Tissue samples were collected from 22 patients with primary neuroblastoma and 9 patients with gangliocytoma treated in Xinhua Hospital Affiliated to Shanghai Jiaotong University School of Medicine from January 2012 to March 2019. All tumor samples from the patients enrolled in the study were surgically removed. The study protocol has been approved by the ethics committee of Xinhua Hospital, and the written informed consent of the participants’ parents or guardians has been obtained.

### Data mining from public databases

The Oncomine (https://www.oncomine.org/) and Tumor Immunity Estimation Resource (TIMER, https://cistrome.shinyapps.io/timer/) database mining tools were used to analyze the expression of MCM6 in different tumors and normal control tissues. Besides, comprehensive information about neuroblastoma-related clinical and prognosis, factors are obtained from R2: Microarray Analysis and Visualization Platform (https://r2.amc.nl) and selected for analysis. We selected three publicly available datasets to analyze tumor prognostic factors, including Kocak (GEO: GSE45547) [17], SEQC (GEO: GSE49710) [18] and Oberthuer (ArrayExpress: E-TABM-38) [19]. All Kaplan-Meier analyses were performed online, and the optimal *p* value and the cutoff value used to separate the high expression group and the low expression group were selected by the median.

### Cell culture and transfection

Human neuroblastoma cell lines (SK-N-BE [2], SK-N-AS, SH-SY5Y) were obtained from ATCC (Manassas, USA), SK-N-SH, IMR-32 were from Chinese Academy of Sciences Cell Bank (Shanghai, China). All of the cells were routinely maintained in a 1:1 mixture of Eagle’s Minimum Essential Medium and Ham’s nutrient mixture F-12 Medium supplemented with 10% fetal bovine serum (Gibco, USA) at 37 °C with 5% CO_2_. The human siRNA MCM6 and the non-targeting siRNA (sequences was in Table S1) were purchased from Riobio Biotechnology (Guangzhou, China). The cells were transfected with siRNA at a final concentration of 60 nM for 24 h performing in Lipofectamine™ RNAimax (Thermo Fisher Scientific, USA). To be specific, cultured the cells in a 6-well plate and performed transfection when the cell density was about 30%. In 200 ul Opti MEM I medium, mixed 0.12 nmol siRNA with 8 μl transfection reagent and incubated for 5 min, added to one well of a 6-well plate, and then add 2 ml complete medium. After 24 h of incubation, the medium was replaced with a standard medium and the cells were ready for further experiments.

### Lentivirus-mediated silence for MCM6

The lentivirus containing shRNA interference oligonucleotide sequence (Table S1) and scrambled control shRNA was constructed and packaged by GeneChem (Shanghai, China). The SK-N-BE [2] cells were infected according to GeneChem’s manufacturer’s protocol. 72 h after infection, 2 μg/ml puromycin (Cat. #ST551, Beyotime, China) was used to screen cells transfected with stable lentivirus. Puromycin-resistant cells were collected 3 days after the addition of puromycin to obtain cells stably transfected with lentivirus.

### Total RNA extraction and quantitative reverse transcription polymerase chain reaction

TRIzol reagent (Invitrogen, USA) was used to extract total RNA from cells. Reverse transcription of the extracted RNA was performed using the PrimeScript™ RT Master Mix (Cat. RR036A, TAKARA, Japan). Then, qRT-PCR was performed on the Applied Biosystems QuantStudio 3 real-time quantitative PCR instrument (Appliedbiosystems, Thermofisher Scientific). The primer sequences in this study are listed in Table S1. GAPDH was used as a standardization control, and the ΔΔCT method was used to calculate relative mRNA expression.

### RNA-seq and data analysis

The Oligo (dT) magnetic beads are used to enrich the mRNA with polyA structure in the total RNA, and the RNA is interrupted to a fragment of about 300 bp in length by means of ion interruption. Using RNA as template, the first strand of cDNA was synthesized with 6-base random primer and reverse transcriptase, and the first strand of cDNA was used as template to construct the second strand of cDNA synthesis library. After that, PCR amplification was used for library fragment enrichment, and then library selection was conducted according to the fragment size, with the library size at 450 bp. The quality of the library was inspected by the Agilent 2100 Bioanalyzer, and then the total concentration of the library and the effective concentration of the library were detected. According to the effective concentration of the library and the amount of data required by the library, the libraries containing different Index sequences (each sample plus a different Index, and finally the off-machine data of each sample are distinguished according to the Index) are mixed in proportion. The mixed library is uniformly diluted to 2 nM, and the single-stranded library is formed by alkali denaturation. After RNA extraction, purification, and library building of the samples, the Next-Generation Sequencing (NGS) is used to perform paired-end (PE) sequencing on these libraries based on the Illumina sequencing platform. The raw sequencing reads can be obtained in the Gene Expression Omnibus (GEO) database with accession number GSE159637.

Analyses were performed in R version 3.5.2. First, filter the raw data, and compare the filtered high-quality sequence (Clean Data) to the reference genome of the species. According to the comparison results, the expression of each gene is calculated. On this basis, further analysis of expression differences, enrichment analysis and cluster analysis were performed on the samples. Compare the Reads on the pair to splice and restore the transcript sequence.

### Western blotting

Whole-cell lysates were harvested for protein analysis. Lysed cells with 8 M urea lysis buffer supplemented with protease inhibitors. Separate proteins by SDS-PAGE gel electrophoresis and transfer to PVDF membrane (Millipore, Sigma Aldrich, USA). The membrane was blocked with 5% BSA at room temperature for 1 h, and then incubated with the appropriate antibody overnight at 4 °C. After washing the blots 3 times with TBST, the membranes were incubated with appropriate HRP-conjugated secondary antibody (Cell Signaling Technology) for 1 h at room temperature, and washed again with TBST. The protein bands were visualized by the Bio-Rad ChemiDoc XRS imaging system. Primary antibody to MCM6 (Cat. ab201683, 1:1000) and β-actin (Cat. #ab8227, 1:1000) were purchased from Abcam. Cyclin D1(Cat. E3P5S, 1:1000), CDK4 (Cat. D9G3E, 1:1000) were purchased from Cell Singling Technology (CST).

### Cell viability assay

Cell viability was analyzed using the Cell Counting Kit-8 (CCK-8) (Yeasen, Shanghai, China). Cells at a density of 3 × 10^3^/well were seeded into 96-well plates and cultured for 4 days. CCK-8 determination was performed every 24 h from the time the cells adhered. Add 100 μl of phenol red-free 1640 medium containing 10% CCK-8 solution to each well and incubate at 37 °C for 2 h. The absorbance value (OD) of each well was measured at 450 nm and 630 nm.

### Colony formation assay

Cells were plated in 6-well culture plates at 100 cells/well. After incubation for 2 weeks at 37 °C, the cells were washed twice with PBS and stained with 0.1% crystal violet solution. The number of colonies containing ≥50 cells was counted under a microscope. The colony formation efficiency was calculated as (number of colonies/number of cells inoculated) × 100%.

### Wound-healing assay

For the wound-healing assay, cells were grown to confluence in a 6-well plate. Artificial wound tracks were created by scraping the confluent cell monolayers with a pipette tip. The cells were fed with serum-free medium. The ability of the cells to migrate into the wound area was assessed every 24 h after scratching.

### Cell migration and invasive assays

Cell motility was assessed by cell migration and invasion assays using transwell chambers with or without Matrigel (Corning). Approximately 1 × 10^6^ cells in medium without FBS were seeded on upper transwell chambers with or without Matrigel and incubated at 37 °C for 48 H*. medium* containing 10% FBS was put in the lower chamber. The invasive cells attached to the lower surface of the membrane insert were fixed, stained using crystal violet (Beyotime, China) and quantified.

### EdU incorporation assay by flow cytometry

For the EdU (5-Ethynyl − 2′- deoxyuridine) incorporation assay, proliferating cells were examined using the Cell-Light EdU Apollo488 In Vitro Flow Cytometry Kit (RiboBio, Guangzhou, China) according to the manufacturer’s protocol. Cells at a density of 1 × 10^6^/well were seeded into 6-well plates and incubated with 50 mM EdU for 3 h. Next, harvested cells and fixed with 4% paraformaldehyde, permeabilized in 0.2% Triton X-100 and staining with Apollo fluorescent dyes. The fluorescence signal at 488 nm was collected by flow cytometer, and the proportion of positive signals labeled with EdU was analyzed by FlowJo software (FlowJo, LLC).

### Cell cycle synchronization

Synchronized SK-N-BE [2] cells in G1 phase were obtained by thymidine-nocodazole sequential blocking method. In short, the cells were cultured to the logarithmic growth phase, thymidine (Cat. #6060, Sigma) was added to the culture medium to a final concentration of 2 mM. After 12 h of culture, cells were washed with PBS, and then added fresh medium with 100 ng/ml nocodazole (Cat. #S2775, Selleck Chemicals). After culturing for 10 h, we shook the culture dish to collect M-phase cells, and the collected M-phase cells were cultured for 3 h to obtain synchronized cells in G1 phase. S phase and G2/M phase synchronized cells were obtained by thymidine double blocking method. That is, added thymidine to the cells in the logarithmic growth phase to a final concentration of 2 mM and cultured for 12 h, then discarded the supernatant, rinsed with PBS, and cultured with fresh medium for 8 h, then added 2 mM thymidine again for 12 h, after all removed thymidine and continue cultured for 2 h to obtain S-phase synchronized cells; and G2/M-phase synchronized cells can be obtained by culturing for 6 h after removing thymidine.

### Cell cycle analysis

The cells in logarithmic growth phase were harvested and seeded into 6-well plates (1 × 10^6^/well) and transfected with si-MCM6 or siRNA control. After 48 h, the cells were collected for flow cytometry. This experiment was repeated three times.

### Mouse xenografts

The subcutaneous model is used for animal research. In the subcutaneous model, 1 × 10 ^6^ SK-N-BE [2] cells were suspended in 0.1 ml PBS medium and transfected with MCM6 shRNA, lentiviral vector and negative control (scrambled) vector. Ten 4–6-week-old male BALB/c nude mice, were obtained from Shanghai Jihui Laboratory Animal Care Co.,Ltd. (Shanghai, China), and randomly divided into two groups (5 mice/group): the LV-shMCM6 group and LV-shNC group. The 2 kinds of stably transfected cells were injected into the armpits of 5 male BALB/c nude mice separately. The mice were maintained in a barrier facility on a rack filtered by HEPA and fed an autoclaved rodent diet. After 45 days, the mice were sacrificed by cutting off necks after anesthesia, and the tumor tissue was surgically removed, weighed and stained with hematoxylin and eosin. All animal handling and procedures have been approved by the Animal Care and Use Committee.

### Statistical analysis

GraphPad Prism 8 (GraphPad Software, Inc. La Jolla, USA) and SPSS version 25.0 software (SPSS, Chicago, IL, USA) for Windows was applied for statistical analysis. The qualitative data were compared with Fisher’s exact test or Pearson’s chi-square test, and the quantitative data were compared with Student’s t test or analysis of variance. The correlations were analyzed by Spearman. Results were expressed as mean ± standard error of the mean (SEM). *p* < 0.05 was considered a statistically significant difference. Significance was expressed as: *p < 0.05, **p < 0.01, and ***p < 0.001.

## Results

### High expression of MCM6 in neuroblastoma is associated with poor pathological classification

**Fig. 1:**
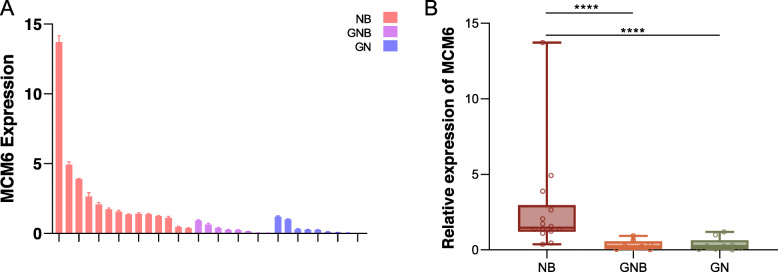
MCM6 mRNA expression in 31 clinical samples. (**A**) The histogram shows the relative expression level of MCM6 mRNA for each sample. (**B**) The samples are divided into 3 categories according to Shimada pathology classification. The mRNA expression of MCM6 in neuroblastoma is significantly higher than that of ganglioeuroblastoma and ganglioneuroma. The value is displayed as the mean ± SEM and statistical significance is expressed as **** *p* < 0.0001. NB, neurobastoma; GNB, ganglioeuroblastoma; GN, ganglioneuroma

We started our research by examining the expression of MCM6 mRNA in 31 neuroblastoma specimens (Fig. 1). The 31 neuroblastoma specimens were divided into three groups according to Shimada pathological classification: neuroblastoma, ganglioblastoma, and ganglioneuroma [20]. One ganglioneuroma sample was set as control, and the relative expression of MCM6 mRNA in each sample was analyzed. 18 samples showed low expression of MCM6 mRNA, and 13 samples showed high expression. Next we evaluated the clinical relevance of MCM6 in pediatric neuroblastoma. We found in this cohort that high expression of MCM6 was positively correlated with worse histopathological typing (Table 1, two-tailed Spearman’s correlation, *r* = 0.671, *p* < 0.0001). Besides, we performed Western blotting on 28 cases of this batch of clinical samples to detect the expression level of MCM6 protein, and found that it was basically consistent with the expression of mRNA (Figure S1).

**Table 1:** The MCM6 mRNA relative expression and clinical information of 31 neuroblastoma tissue samples

	MCM6 expression		Total	***P*** value
	Low	High		
**Sex**				0.071
Male	8	10	18	
Female	10	3	13	
**Age at diagnosis**				0.008
≤ 18 m	1	6	7	
> 18 m	17	7	24	
**Stage**				0.484
I, II, IVs	2	4	6	
III, IV	8	8	16	
**Primary site**				0.768
Retroperitoneum	5	3	10	
Postmediastinum	13	10	21	
**Hisopathology diagnosis**				0.000
GN	8	1	9	
GNB	8	0	8	
NB	2	12	14	
**Bone marrow metastasis**				0.841
Positive	2	1	3	
Negative	17	11	28	
**Risk**				0.396
Low	11	5	16	
Mid	3	1	4	
High	5	6	11	

The value is displayed as the number of cases. *NB* neurobastoma, *GNB* ganglioeuroblastoma, *GN* ganglioneuroma

### MCM6 is a potential prognostic factor in neuroblastoma

**Fig. 2:**
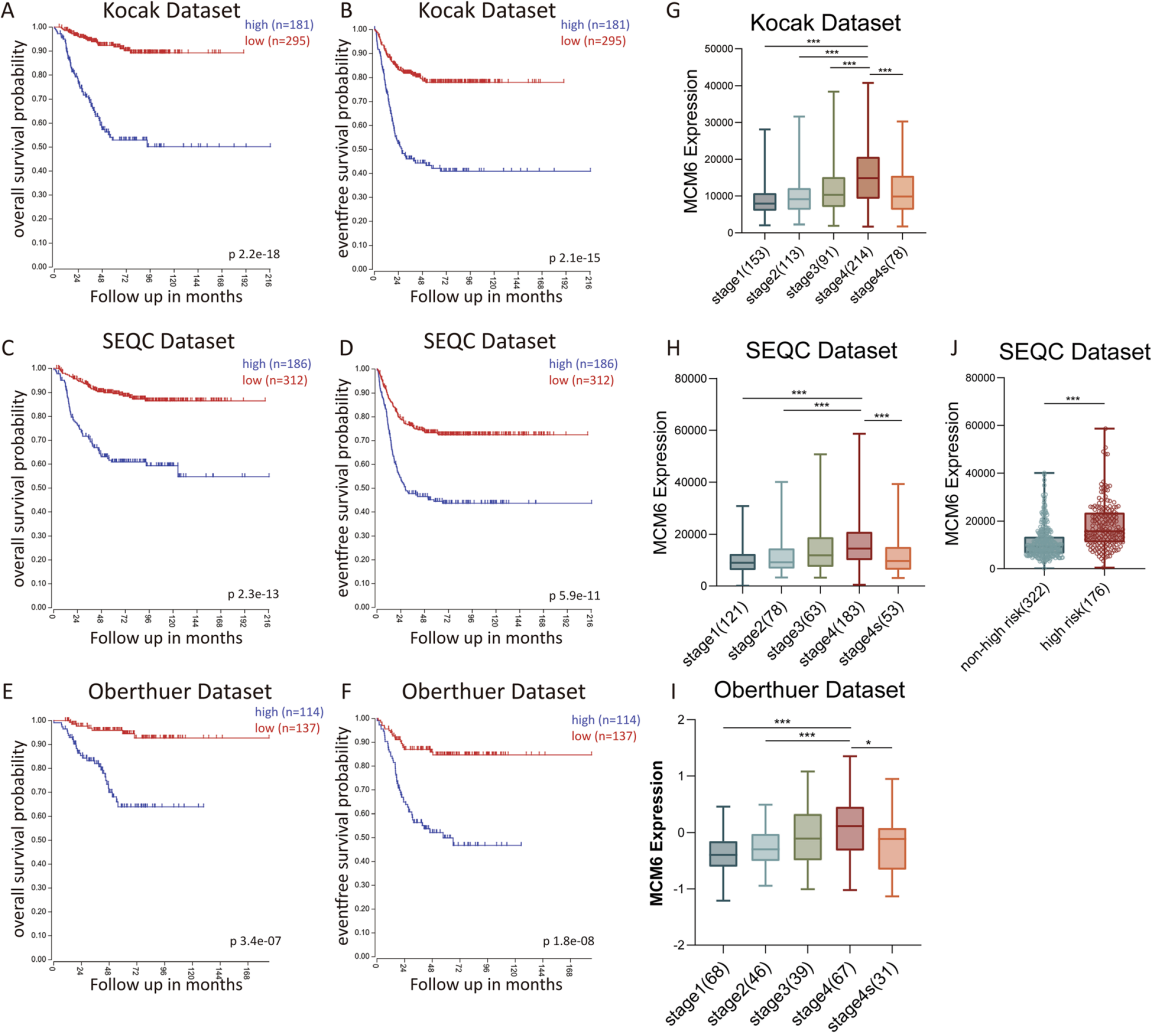
The prognostic value of MCM6 in three validated clinical neuroblastoma datasets and the value of tumor INSS staging and risk classification. (**A**-**B**) Kaplan-Meier analysis of OS and EFS based on the MCM6 expression in Kocak dataset, and the log-rank test *P* value is displayed (*n* = 476, 173 patients without survival information were not included in the analysis); (**C**-**D**) Kaplan-Meier analysis of OS and EFS based on the MCM6 expression in SEQC dataset, and the log-rank test P value is displayed (*n* = 498); (**E**-**F**) Kaplan-Meier analysis of OS and EFS based on the MCM6 expression in Oberthuer dataset, and the log-rank test P value is displayed (*n* = 251); (**G**-**I**) The box plot shows the expression level of MCM6 in the above three public datasets in different INSS stages (stage 1, 2, 3, 4, 4 s); (**J**) The box plot shows the expression level of MCM6 in the high-risk group and low-risk group of neuroblastoma in the SEQC data set. Since the risk classification data were not included in the Kocak and Oberthuer datasets, no relevant analysis was performed. The value is displayed as the mean ± SEM and statistical significance is expressed as* *p* < 0.05, *** *p* < 0.001

Through the mining of Oncomine database and TIMER database, we found that MCM6 expression is higher in most cancers, such as sarcoma, colorectal cancer, lung cancer, cervical cancer, and liver cancer (Figure S2). In order to further evaluate and confirm the possibility of MCM6 as a prognostic marker in neuroblastoma, we performed Kaplan-Meier overall survival (OS) and event-free survival (EFS) analysis using three different tumor neuroblastoma public datasets through the online R2: microarray analysis acquisition and visualization platform. In the Kocak, SEQC and Oberthuer datasets, neuroblastoma patients with high MCM6 expression all showed poorer OS and EFS than low MCM6 expression cohorts (Fig. 2A-F). Moreover, the expression of MCM6 is different in different INSS tumor stages. Compared with stage 1 and 2, the expression in stage 4 tumors is significantly increased (Fig. 2G-I). In addition, we analyzed MCM6 expression in neuroblastoma of high-risk group and low-risk group in the SEQC dataset, and found that the expression of MCM6 in neuroblastoma in the high-risk group was significantly higher than that in the low-risk group (*p* < 0.001) (Fig. 2J). In summary, our data show that high expression of MCM6 significantly associates with poor prognosis of neuroblastoma.

### Knockdown of MCM6 suppresses neuroblastoma cell proliferation, migration and invasion in vitro

**Fig. 3:**
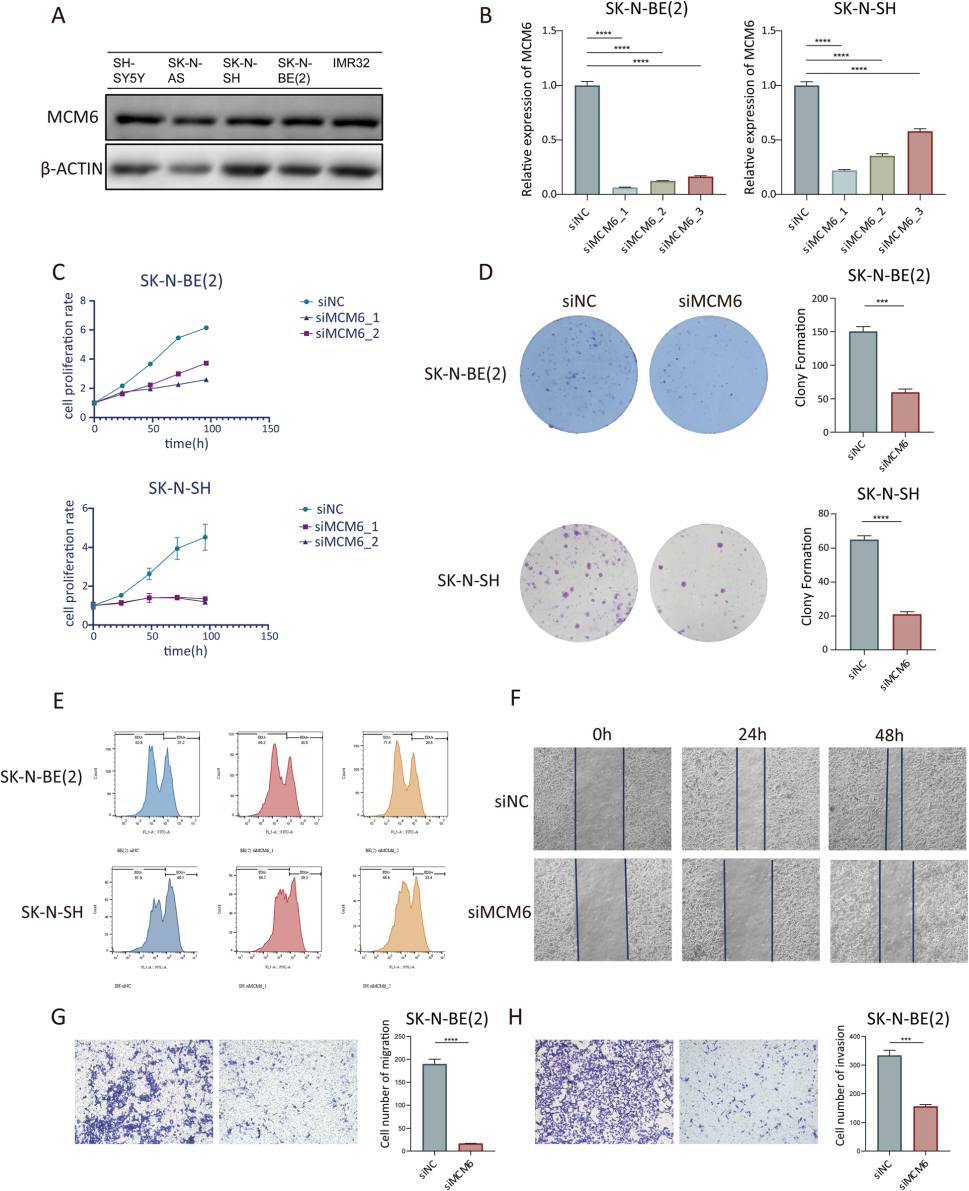
Functional experiment of MCM6 in neuroblastoma cells. (**A**) Western blotting verified the protein expression of MCM6 in 5 neuroblastoma cell lines; (**B**) Interference efficiency of 3 small interfering RNAs with different sequences on MCM6, detected by qRT-PCR; The in vitro proliferation function of MCM6 was measured by CCK-8 (**C**), colony formation (**D**) and flow cytometry EdU labeling detection (**E**). The wound healing experiment (**F**) and the Transwell migration and invasion experiment (**G**-**H**) verified the migration (**G**) and invasion (**H**) activities of MCM6 on neuroblastoma cells in vitro. EdU, 5-Ethynyl − 2′- deoxyuridine

In vitro experiments show that MCM6 is expressed at a higher level in neuroblastoma cell lines (Fig. 3A). In order to comprehend the function of MCM6 in neuroblastoma cells, we designed three siRNAs (siRNA1, siRNA2 and siRNA3) to silence the expression of MCM6. After qRT-PCR (Student’s t-test, *p* < 0.05) and western blotting, SK-N-BE [2] and SK-N-SH cells treated with siRNA decreased the expression of MCM6 by more than 70% compared with the control group. SiRNA1 and siRNA2 have the best silencing effect, so they were chosen for further research (Fig. 3B).

We examined the effect of MCM6 expression on the growth of neuroblastoma cells in vitro. Using CCK-8 analysis (Fig. 3C), colony formation assay (Fig. 3D) and EdU incorporation analysis (Fig. 3E), we found that knocking down MCM6 significantly inhibited the cell proliferation of SK-N-BE [2] and SK-N-SH cells. After MCM6 knockdown, through CCK-8 detection, we found that the 96 h proliferation inhibition rate of SK-N-BE [2] cells was 57.7%, and the inhibition rate in SK-N-SH cells reached 73.5%. Besides, we found that SK-N-BE [2] cells with positive EdU labeling after MCM6 knockdown decreased by 8.7% and SK-N-SH cells decreased by 14.7% compared with the NC group. In the colony formation experiment, SK-N-BE [2] cells in the NC group formed an average of 150.3 colonies, while in the MCM6 knockdown group formed an average of 59.7 colonies. In SK-N-SH cells, the number of colonies formed in the NC and MCM6 knockdown groups were 65 and 21, respectively. At the same time, wound healing and Matrigel-coated (for invasion) or-uncoated (for migration) Transwell analysis showed that MCM6 knockout effectively inhibited the invasion and metastasis of neuroblastoma (Fig. 3F-G). In the wound healing experiment, the healing rate of the cells in the NC group exceeded 70% at 48 h after the scratch, while the healing rate of the MCM6 knockdown group was lower than 30%. In the Transwell migration experiment, the number of migrating cells in the NC group was 189.7 /HPF, while the number of migrating cells in the MCM6 knockdown group was 16.7 /HPF. In the Transwell invasion experiment, the number of migrating cells in the NC group was 333.7 /HPF, and the number of migratory cells in the MCM6 knockdown group was 155.3 /HPF. These results reveal the role of MCM6 in promoting the progression of neuroblastoma *in ​​vitro*.

### Inhibition of MCM6 expression in a mouse model can inhibit the growth of neuroblastoma cells in vivo

**Fig. 4:**
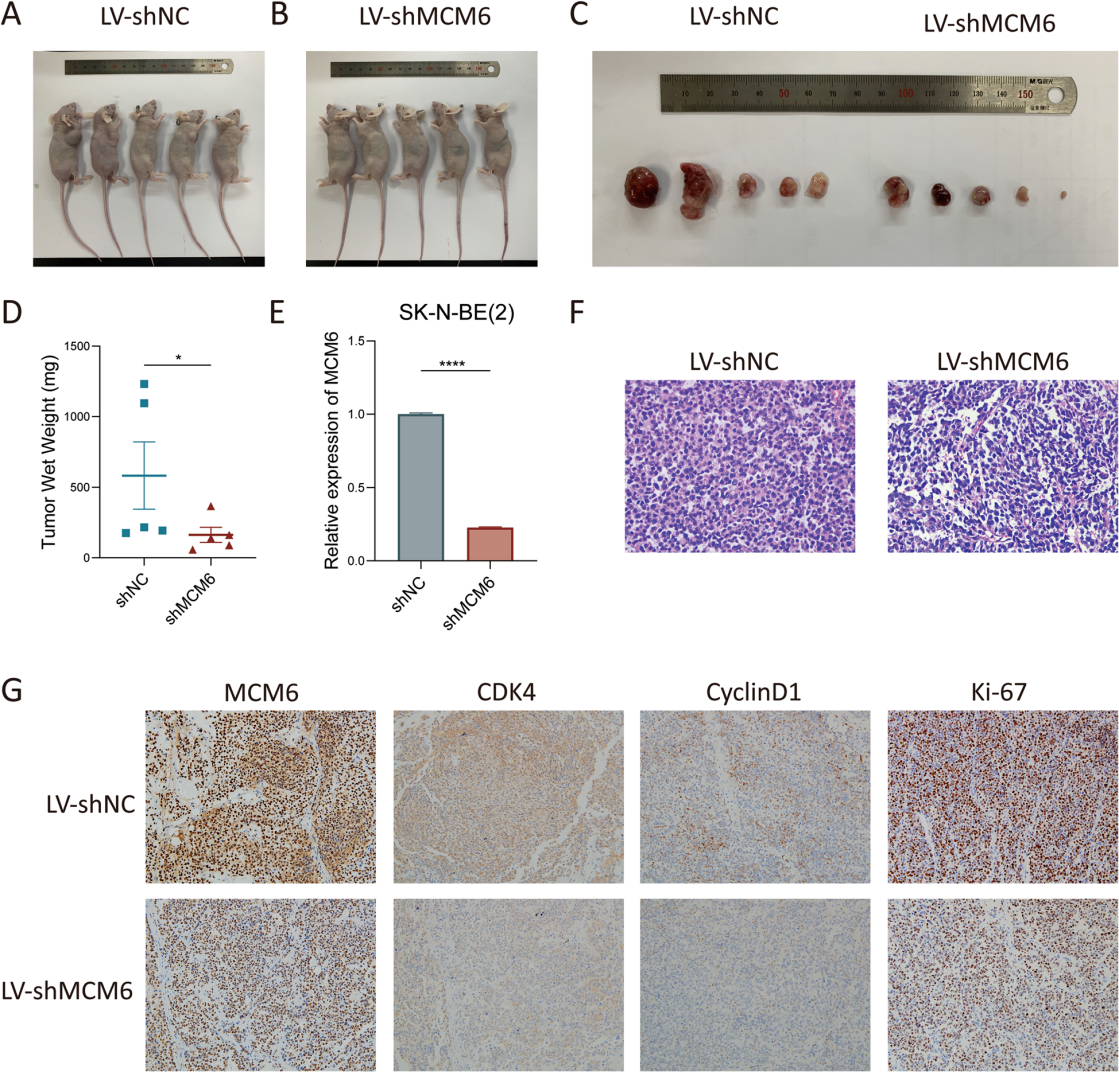
MCM6 knockdown inhibited the growth of neuroblastoma in vivo. (**A**-**B**) BLAB/c nude mice were sacrificed 45 days after subcutaneous injection and the tumor was dissected. (**D**) The wet weight of tumors formed by MCM6 knockdown cells were significantly smaller than that of the control (*p* < 0.05). (**E**) The knockdown efficiency of the lentivirus tool on MCM6 in SK-N-BE [2] cells reached more than 75% (*p* < 0.0001). (**F**) H&E staining of subcutaneous tumors in BALB/c nude mice (× 400). (**G**) Immunohistochemical staining of subcutaneous tumors in BALB/c nude mice on target of MCM6, CDK4, CyclinD1 and Ki-67 separately (× 200). The value is displayed as the mean ± SEM and statistical significance is expressed as *p < 0.05, **** *p* < 0.0001

In order to explore the role of MCM6 in the ability of neuroblastoma tumorigenesis in vivo, we established the shMCM6 SK-N-BE [2] stable cell line (LV-shMCM6) to study its biological functions in a mouse model. We initiated tumor growth by subcutaneously injecting 1 × 10 ^6^ LV-shMCM6 cells into BALB/c mice, and monitored tumor growth by measuring the size. Forty-five days after injection, we observed that tumors of LV-shMCM6 mice were significantly smaller than those of mice that received empty vector (LV-shNC) (Fig. 4A-C). Consistent with this finding, the tumor weight of LV-shMCM6 mice is lighter than that of LV-shNC mice (Fig. 4D). H&E staining shows that tumors in LV-shNC mice have more vigorous mitotic figures than LV-shMCM6 mice (Fig. 4F). Immunohistochemical staining for cell cycle and proliferation indicators shows that the reduced expression of CyclinD1, CDK4 and Ki-67 in the subcutaneous tumors in the MCM6 knockdown group (Fig. 4G). These results indicate that MCM6 is critical to the growth of neuroblastoma tumors.

### Gene expression regulation associated with MCM6 knockdown

**Fig. 5:**
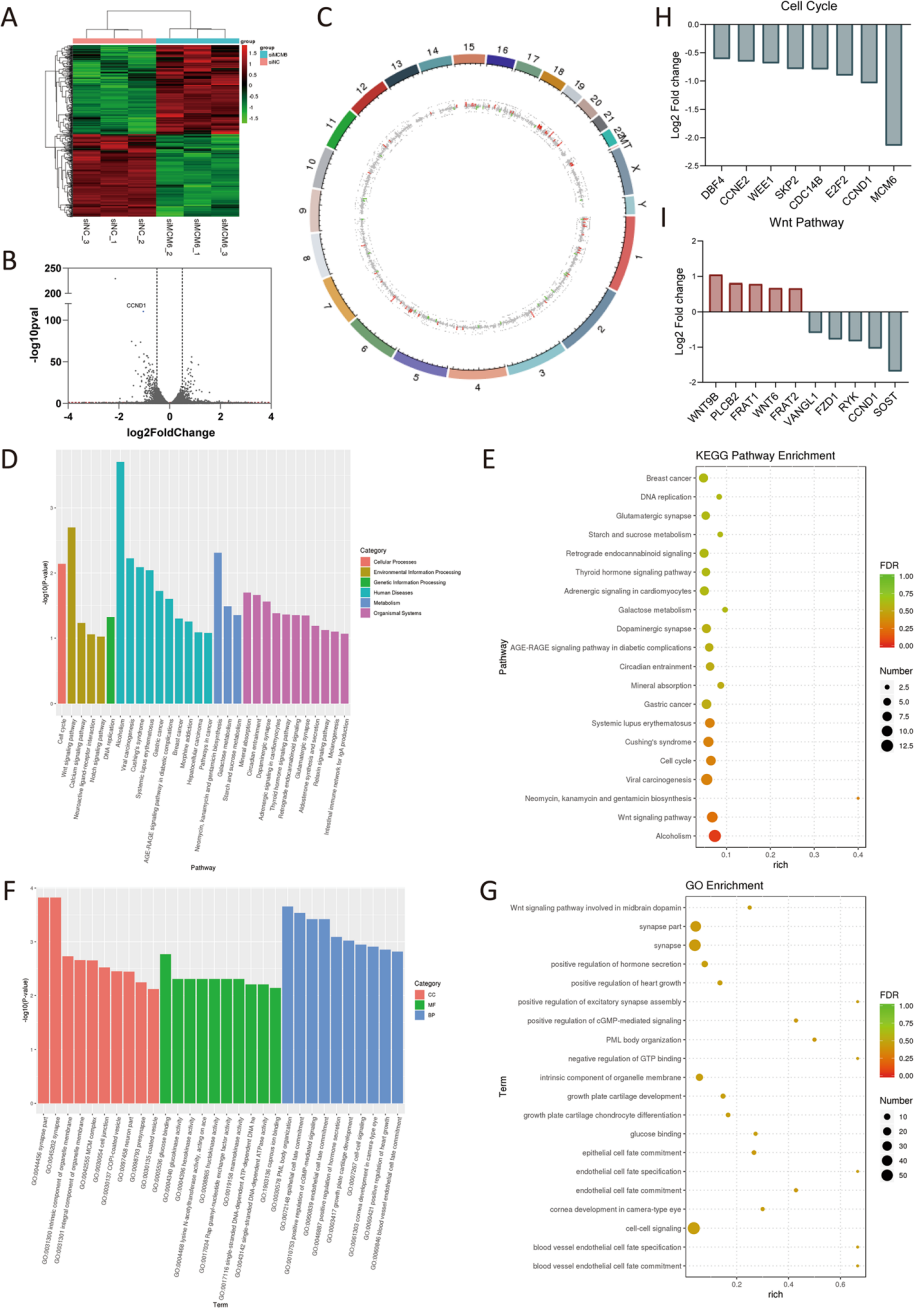
MCM6 activates pathways related to cell cycle regulation. (**A**) Cluster heat map of 6 samples. We used the R language Pheatmap software package to perform two-way clustering analysis on the union of different genes and samples of all comparison groups, clustering according to the expression level of the same gene in different samples and the expression pattern of different genes in the same sample, using the Euclidean method calculate the distance, the longest distance method of hierarchical clustering (complete Linkage) for clustering. In the figure, genes are represented horizontally, and each column is a sample. Red represents high expressed genes and green represents low expressed genes. (**B**) The volcanic map was drawn according to the gene distribution, gene expression fold change and significance results. On the left was the down-regulated gene of siMCM6 group relative to siNC group, and on the right was the up-regulated gene. The two vertical dotted lines were the threshold of 1.5 times fold change of gene expression difference, and the horizonal dotted line is the threshold of *p* value =0.05. (**C**) Genome circle diagram. We used the R language Circlize package to mark the differentially expressed RNA on the genome according to the genome information and the results of RNA differential expression analysis. The outermost circle is the chromosome band, from the outside to the inside are the differential expression analysis results of different differential analysis. Red and green are the histograms of the log2FoldChange values ​​of the up-regulated and down-regulated genes, and the gray is the scatter plot of the log2FoldChange values ​​of the undifferentially expressed genes. (**D**-**E**) The GO enrichment analysis results of differentially expressed genes. GO classification is carried out based on MF, BP and CC, and the top 10 GOs with the smallest *p* value, as well as the most significant enrichment in each GO classification term were selected to entry for display (**D**). The enrichment degree is measured by Rich factor, FDR value and the number of genes enriched to this GO Term. Among them, Rich factor refers to the ratio of the number of differential genes enriched in the GO Term to the number of annotated genes. The greater the Rich factor, the greater the degree of enrichment. FDR generally ranges from 0 to 1, the closer to 0, the more significant the enrichment. Select the top 20 GO Term entries with the smallest FDR value, that is, the most significant enrichment, for display (**E**). (**F**-**G**) The KEGG enrichment analysis results of differentially expressed genes. Select the top 20 pathways with the smallest *p* value for display (**F**). The degree of enrichment is measured by Rich factor, FDR value and the number of genes enriched on this pathway. As well as GO enrichment analysis, we selected the top 20 KEGG pathways with the smallest FDR value namely the most significant enrichment, for display (**G**). (**H**) Differentially expressed genes of the enriched cell cycle pathways and their differential expression levels. (**I**) Differentially expressed genes of the enriched Wnt pathways and their differential expression levels. GO, Gene Ontology; KEGG, Kyoto Encyclopedia of Genes and Genomes; FDR, *p* value correction; MF, molecular function; BP, biological process; CC, cell component

To identify potential targets regulated by MCM6 in neuroblastoma, we performed RNA-seq in SK-N-BE [2] cell line (Fig. 5A-G). According to the different expression fold change = 1.5, we found that the expressed levels of 442 genes have changed, of which 228 genes were up-regulated and 214 genes were down-regulated (Fig. 5A-B). Differentially expressed genes are mainly distributed on chromosomes 1, 2, 5, 6, 15, 16, 19 (Fig. 5C). KEGG pathway analysis further revealed that the differentially expressed genes are mainly concentrated in cell cycle, Wnt signaling pathway, DNA replication, et al. (Fig. 5D-E). In addition, GO analysis showed that the differentially expressed genes are the most abundant in synapse, cell-cell signaling, intrinsic component of organelle membrane, et al. (Fig. 4F-G). In the cluster analysis of GO (Fig. 5D-E) and KEGG (Fig. 5F-G), it was found that differential expression of genes related to the cell cycle, including cell cycle, cell cycle checkpoint, G1/S transition of mitotic cell cycle, cell cycle process, cell cycle G1/S phase transition, cyclin-dependent protein serine/threonine kinase regulator activity and regulation of response to cell cycle checkpoint signaling (*p* < 0.05). The specific differentially expressed genes related to cell cycle pathways and their expression levels are shown in Fig. 5H, where the differential expression of CCND1 is the most prominent.

### Cell cycle arrest induced by suppression of MCM6 expression

**Fig. 6:**
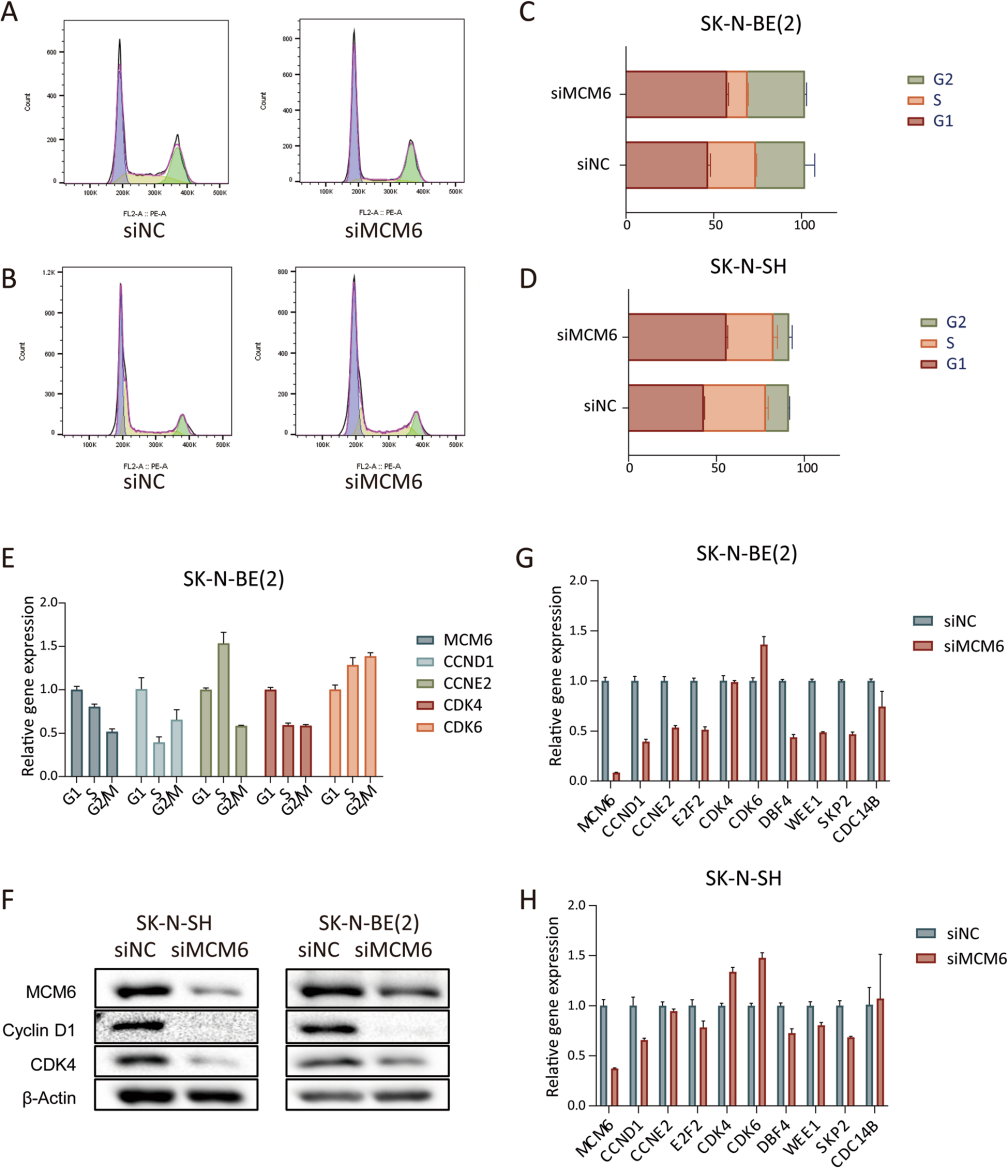
MCM6 can regulate the cell cycle of neuroblastoma at both the cell and protein level. (**A**-**D**) Cell cycle analysis was performed on neuroblastoma cell lines using flow cytometry. After MCM6 silenced, SK-N-BE [2] and SK-N-SH cells both showed an increase in G1 phase components and a decrease in S phase components. (**E**) The expression of cell cycle regulated genes in different cell cycle phases of SK-N-BE [2] cells. (**F**) Verify the expression of cell cycle regulation related proteins by WB. The expression of CDK4 and CyclinD1 was down-regulated after MCM6 silence. (**G**-**H**) According to the cell cycle regulation pathway enriched by RNA-seq, the relevant genes on the pathway were verified by qPCR. The value is displayed as the mean ± SEM and statistical significance is expressed as **** *p* < 0.0001

Taking into account the findings of the above assay, we further studied the effect of MCM6 on the cell cycle of neuroblastoma SK-N-BE [2] and SK-N-SH cell lines. Compared with the control group, the proportion of cells in the G1 phase of the siMCM6 group increased significantly (the proportion of SK-N-BE [2] cells in the G1 phase increased by an average of 10.8%, and the proportion of SK-N-SH cells in the G1 phase increased by an average of 12.9%) while the proportion of cells in the S phase decreased significantly (the proportion of SK-N-BE [2] cells in the S phase is reduced by an average of 15.5%, and the proportion of cells in the S phase of SK-N-SH is reduced by an average of 8.6%) (Fig. 6A-D). This indicates that cells in the siMCM6 group stagnated in the G1 phase and failed to enter the S phase. We synchronized SK-N-BE [2] cells in the G1, S and G2/M phases of the cell cycle respectively, and detected the expression of MCM6 and cell cycle regulatory genes in different phases. We found that the expression of MCM6 increased in the G1 phase and decreased in the S phase, and the expression of CCND1 and CDK4 also showed similar characteristics. In terms of mechanism, the content of CyclinD1 and CDK4 is lower in cells treated with siMCM6 (Fig. 6E-F), which indicates that inhibiting MCM6 can delay the development of cell cycle G1/S by down-regulating the expression of cell cycle checkpoint proteins.

## Discussion

Neuroblastoma, as the most common extracranial solid tumor in children, has a high morbidity and mortality rate, but it is also highly heterogeneous. Course and outcome of neuroblastoma disease is very diverse. Some children’s tumors can naturally regress or mature, but there are also quite a few children with metastasis and recurrence. For relapsed and refractory neuroblastoma, there is currently a lack of effective specific drugs, while powerful chemotherapeutics can cause many adverse reactions. Therefore, the exploration of potential therapeutic targets for neuroblastoma has always attracted the attention of researchers.

Gene amplification and overexpression of MCM6 are found in many human cancers [12–15], which correlates with tumor genesis and progress, as well as aggressive biological behaviors. In this study, we provided evidence that MCM6 is a potential novel therapeutic target for neuroblastoma patients. The recurrence, invasion and metastasis of neuroblastoma are the reasons for the poor long-term survival of neuroblastoma patients. Early diagnosis and active intervention may bring significant survival benefits for those patients. Analysis of clinical significance showed that high expression of MCM6 is related to the progression and high risk of neuroblastoma. In addition, patients with increased MCM6 expression have poorer survival and a higher cumulative recurrence rate. Through in vitro experiments, we found that MCM6 silencing can significantly inhibit neuroblastoma cell proliferation, migration and invasion. Moreover, the in vivo experiments also confirmed the important role of MCM6 in tumor genesis, and suggested that this effect is related to cell proliferation. All these results indicate that MCM6 may play an important role in the development of neuroblastoma.

During the enrichment analysis of RNA-seq results, we also found significant differential expression of Wnt Pathway related genes. Among them, WNT6 and WNT9B were significantly up-regulated after MCM6 interference. Wnt Pathway plays an important role in embryogenesis and tumor development [21]. Wnt pathway includes the classic Wnt/β-catenin pathway and the non-canonical Wnt/Ca^2+^ pathway and Wnt/PCP pathway [22]. Multiple dysfunctions and mutation of Wnt/β-catenin signaling pathway have been reported to be related to the promotion of cell proliferation and metastasis in various tumors, including hepatoma [23], colon carcinoma [24] and leukemia [25]. In in vitro experiments, we found that MCM6 promotes the migration and invasion of neuroblastoma cells, which were consistent with those reports. Therefore, we boldly speculate that the activation of Wnt/β-catenin signaling pathway is an important mechanism for MCM6 to induce neuroblastoma metastasis. Of course, it is also possible that the migration is a secondary effect given that the major effect of MCM6 on cell proliferation.

We found that the neuroblastoma cell cycle was blocked in G1/S after the inhibition of MCM6 expression. The effect of MCM complex on the cell cycle has also been reported in a variety of tumors [26–28]. After interfering expression of MCM6, the neuroblastoma cells from G1 to S phase are blocked, which means that the process of DNA replication is disturbed. The corresponding cyclin protein expression changes were confirmed at the mRNA and protein levels, respectively. In the classic cell cycle theory, to enter the cell cycle and start DNA replication, the cell must pass through strictly regulated restriction points from G1 to S phase [29], which is largely regulated by CDK4/6 checkpoint proteins in the G1/S phase [30]. The downregulation of CDK4/6 after MCM6 interference also supports that the G1/S transition is blocked. Our results indicate that MCM6 is involved in cell cycle. Thereby promotes tumor cell proliferation and poor tumor characterization The disorder of the cell cycle promotes the instability of the genome and leads to tumor apoptosis [31]. Therefore, we believe that MCM6 may be a promising neuroblastoma biomarker.

## Conclusion

MCM6 acts as a tumor promoter by activating CDK4-Cyclin D1 signaling, and subsequently regulates cell cycle progression in G1/S phase in neuroblastoma. This study proved the importance of the expression of MCM6 in neuroblastoma, and MCM6 may be a new prognostic indicator and a potential novel therapeutic target of neuroblastoma patients.

## Supplementary Information

**Additional file 1: Table S1.**

**Additional file 2: Figure S1.** MCM6 protein expression in 28 clinical samples. The tissue source is consistent with the tissue used to detect MCM6 mRNA expression in Figure 1. As the sample is not enough to extract protein, 2 cases of neuroblastoma and 1 case of ganglioneuroma are missing. NB, neurobastoma; GNB, ganglioeuroblastoma; GN, ganglioneuroma. **Figure S2.** We used public datasets to analyze the expression of MCM6 in tumors and control normal tissues. The MCM6 mRNA levels were analyzed using Oncomine database with *P*-value of 0.01, fold change of 2, and gene ranking of all. The analysis showed that compared with normal tissues, MCM6 expression is higher in most cancers, such as sarcoma, colorectal cancer, lung cancer, cervical cancer, and liver cancer (Figure S1A). Data from Tumor Immunity Estimation Resource also showed that the expression of MCM6 in almost all TCGA tumors was significantly higher than that in neighboring normal tissues (Figure S1B). The results suggest that MCM6 is a promising tumor prediction and treatment target.

**Additional file 3.**

## Data Availability

The datasets generated during the current study is available in the following repository: GEO: GSE159637, https://www.ncbi.nlm.nih.gov/geo/query/acc.cgi?acc=GSE159637 The datasets analysed during the current study are available in the following repositories: Oncomine (https://www.oncomine.org/). Tumor Immunity Estimation Resource database (TIMER, https://cistrome.shinyapps.io/timer/). Kocak (GEO: GSE45547, https://www.ncbi.nlm.nih.gov/geo/query/acc.cgi?acc=GSE45547). SEQC (GEO: GSE49710, https://www.ncbi.nlm.nih.gov/geo/query/acc.cgi?acc=GSE49710). Oberthuer (ArrayExpress: E-TABM-38, https://www.ebi.ac.uk/arrayexpress/experiments/E-TABM-38/).

## Abbreviations

MCM: Minichromosome maintenance complex component
NB: Neuroblastoma
GNB: Ganglioeuroblastoma
GN: Ganglioneuroma
OS: Overall survival
EFS: Event-free survival
HPF: High power field
HEPA: High Efficiency Particulate Air
GO: Gene Ontology
KEGG: Kyoto Encyclopedia of Genes and Genomes
CDK: Cyclin-dependent kinase
