# Supplementary figures and images for: MCM6 indicates adverse tumor features and poor outcomes and promotes G1/S cell cycle progression in neuroblastoma
Source: BMC Cancer. 2021 Jul 7;21:784. doi: 10.1186/s12885-021-08344-z (PMC8262023; doi:10.1186/s12885-021-08344-z)

## Slide 1
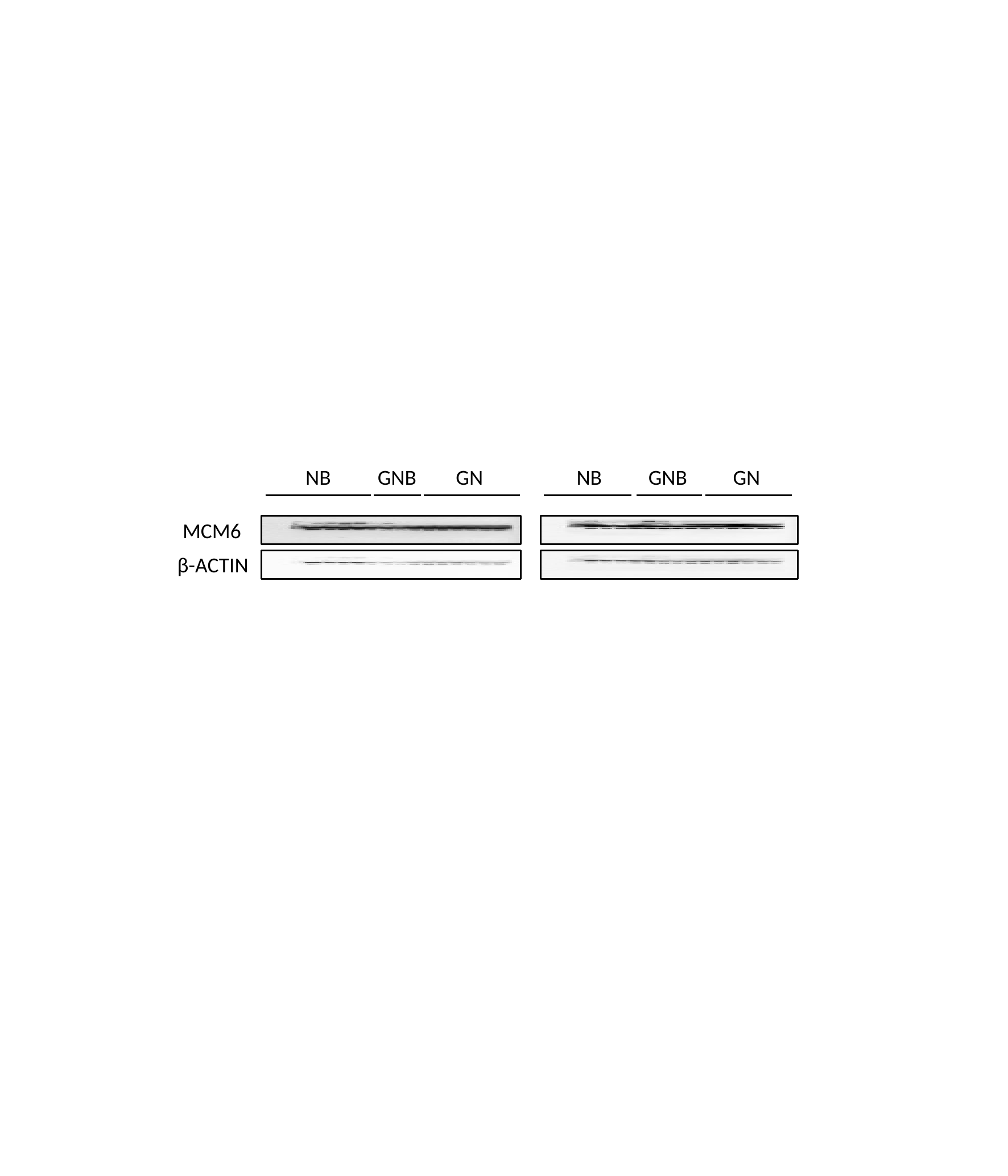

NB
GNB
GN
NB
GNB
GN
MCM6
β-ACTIN

## Slide 2
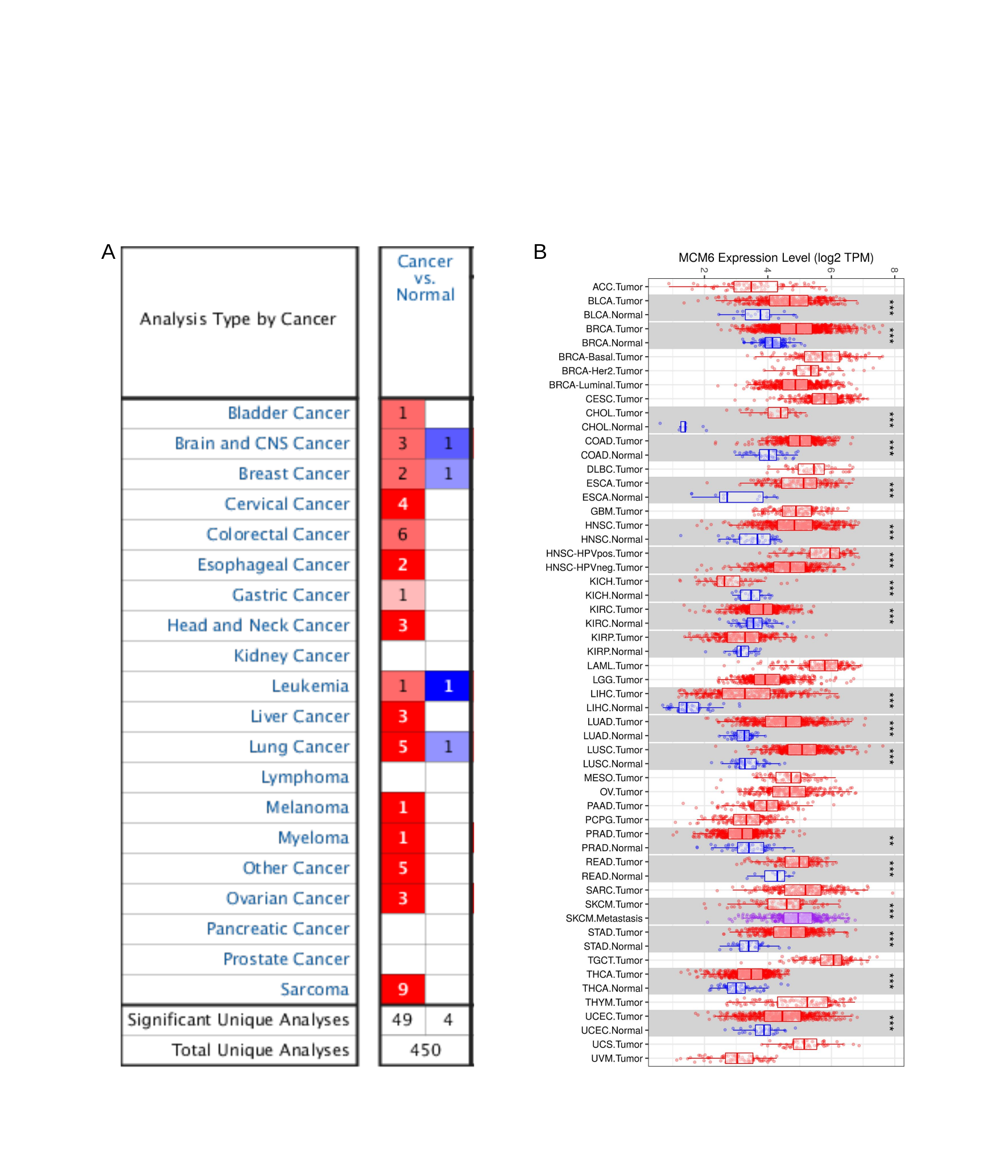

A
B

Supplement: Supplementary file 2 — Additional file 2: Figure S1. MCM6 protein expression in 28 clinical samples. The tissue source is consistent with the tissue used to detect MCM6 mRNA expression in Figure 1. As the sample is not enough to extract protein, 2 cases of neuroblastoma and 1 case of ganglioneuroma are missing. NB, neurobastoma; GNB, ganglioeuroblastoma; GN, ganglioneuroma. Figure S2. We used public datasets to analyze the expression of MCM6 in tumors and control normal tissues. The MCM6 mRNA levels were analyzed using Oncomine database with P-value of 0.01, fold change of 2, and gene ranking of all. The analysis showed that compared with normal tissues, MCM6 expression is higher in most cancers, such as sarcoma, colorectal cancer, lung cancer, cervical cancer, and liver cancer (Figure S1A). Data from Tumor Immunity Estimation Resource also showed that the expression of MCM6 in almost all TCGA tumors was significantly higher than that in neighboring normal tissues (Figure S1B). The results suggest that MCM6 is a promising tumor prediction and treatment target. [file 12885_2021_8344_MOESM2_ESM.pptx]
